# Genome-Wide Identification and Transcriptome Analysis of the Hsp70 Gene Family in *Monodonta labio* Reveals Its Role in Response to Nanoplastics Stress

**DOI:** 10.3390/genes15030291

**Published:** 2024-02-25

**Authors:** Jingjing He, Xiaolu Han, Shaolei Sun, Shihuai Jin, Mengyuan Liu, Zhiqiang Han

**Affiliations:** 1 Fishery College, Zhejiang Ocean University, Zhoushan 316022, China; hejingjing@zjou.edu.cn (J.H.); hxl1014@163.com (X.H.); ssl_0207@163.com (S.S.); 15165669923@163.com (M.L.); 2 College of Plant Protection, Fujian Agriculture and Forestry University, Fuzhou 350002, China; jinshihuai@163.com

**Keywords:** *Monodonta labio*, heat shock protein 70, nanoplastics stress, phylogeny, gene duplication, expression pattern

## Abstract

For marine invertebrates, the disruption of organismal physiology and behavior by nanoplastics (NPs) has been extensively reported. Heat shock proteins (Hsps) are important for redundant protein breakdown, environmental changes, and intracellular protein transport. An exhaustive identification of Hsp70 genes and an experiment where different concentrations of NPs were stressed were performed to study how Hsp70 genes respond to NPs stress in *Monodonta labio*. Our results identified 15 members of Hsp70 within the genome of *M. labio* and provided insights into their responses to different concentrations of acute NP stress. Phylogenetic analyses revealed extensive amplification of the Hsp70 genes from the Hsc70 subfamily, with gene duplication events. As a result of NP stress, five of fifteen genes showed significant upregulation or downregulation. Three Hsp70 genes were highly expressed at an NP concentration of 0.1 mg/L, and no genes were downregulated. At 10 mg/L, they showed significant upregulation of two genes and significant downregulation of two genes. At 1 mg/L treatment, three genes were significantly downregulated, and no genes were significantly upregulated. Moreover, a purifying selection was revealed using a selection test conducted on duplicate gene pairs, indicating functional redundancy. This work is the first thorough examination of the Hsp70s in Archaeogastropoda. The findings improve knowledge of Hsp70s in molluscan adaptation to NP stress and intertidal living and offer essential data for the biological study of *M. labio*.

## 1. Introduction

Cells can be protected from stress by heat shock proteins (Hsps) that have been conserved throughout evolution [1]. Hsp70 participates in a number of post-stress mechanisms, and under normal physiological conditions, including adaptation to stress [2], development [3], and apoptosis [4], because of its chaperone properties. Through its interactions with unfolded proteins, Hsp70 inhibits irreversible protein aggregation and catalyzes substrate refolding in a way that is reliant on co-chaperone molecules and ATP [2]. When the cell is subjected to stresses, such as heat, salinity, ultraviolet light, heavy metals, and various chemicals, Hsps are expressed rapidly to provide a potent buffering system to adapt to such stresses [5].

Following the identification of Hsps in *Drosophila*, researchers started examining the composition and capabilities of these proteins [3]. Multiple Hsp70 gene products exist in all eukaryotes, and they vary from one another in terms of gene structure, subcellular location, and expression level [6]. Hsp genes, particularly those in the Hsp70 family, are very conserved. Hsps are currently divided into multiple categories according to their function, amino acid sequence, and molecular weight [7].

*Monodonta labio* (Linnaeus, 1758) is an important fishery commercial mollusk that belongs to the family Trochidae (Mollusca, Gastropoda) [8]. It is widely distributed in the Indian Ocean–Pacific Ocean and inhabits the intertidal zone [8]. It lives in a variety of intertidal environments, including mangroves and rocky, cobble, and boulder shorelines [9]. *M. labio* is a remarkable component of the intertidal zone and a eurythermic and eurysalinity marine mollusc; it is very tolerant to a broad range of temperatures (0 °C–28 °C) and salinity conditions (13.23–34.95 psu).

Nanoplastics (NPs) pose a threat to the growth and survival, feeding activities, embryogenesis, immune systems, fecundity, and metabolisms of marine organisms and thus have attracted great attention [10]. Since *M. labio* feeds on seaweed and NPs are attached to seaweed, the species is susceptible to NPs [11]. *M. labio* is consistently found in intertidal zones, which are in close contact with NPs, making it a prevalent medium for NPs to enter the human body [12]. In order to prevent damage and preserve cellular homeostasis, the evolution of organisms can activate some genes that adapt to stress, such as Hsp70s; however, little is known about how abiotic stress influences Hsp70s’ expression in the marine environment [13]. The shells of mollusks provide excellent models for researching adaptation evolution and plasticity. Therefore, *M. labio* has been widely used as a model for the adaptation of the abiotic stress of Mollusca [11]. However, genes and pathways that may be involved in this defense response have not yet been discovered, and the mechanism underpinning the molecular toxicology to abiotic stress is yet unknown. To evaluate the impact of microplastics on mollusks and comprehend the biological role of Hsp70s in molluscan adaptation to NP stress and intertidal lifestyle, ecotoxicology studies ought to be carried out.

The Hsp70s has so far been systematically found in mammals, fishes [14,15], crustaceans [16], Echinozoa [17], and bivalves [18]. The exploration of Hsp70s in humans and *Larimichthys* crocea has unveiled a total of 17 members [15,19]. Phylogenetic analyses and systematic identification of the Hsp70s were conducted in bivalves, for example *Patinopecten yessoensis* and *Crassostrea gigas* [18,20]. The Hsp70s expanded; 73 of 86 *hsp70* genes were found to be expanded in Pacific oysters, and 56 of 61 *hsp70* genes were expanded in scallops. Adaptation of oysters to a high-stress intertidal-fixation life may be primarily due to an increase in *hsp70* genes [21]. Tandem duplication of *hsp70* genes may have evolved adaptively to produce toxic stress responses [20]. Nevertheless, no research has been conducted on Hsp70s in snails. Therefore, further investigation is needed to identify and analyze the evolution of Hsp70s across the snail genome.

To comprehend the changes in Hsp70 genes’ expression and its function during acute NP exposure, the *hsp70* gene was discovered and examined using bioinformatics techniques. The findings of this investigation can offer fundamental information for the biological analysis of *M. labio,* as well as the molecular regulation of the intertidal snails’ response to NP stress.

## 2. Materials and Methods

### 2.1. Identification of the Hsp70 Gene Family

The National Genomics Data Center database (https://ngdc.cncb.ac.cn/) (accessed on 11 December 2022) provided the genome data that were downloaded. We downloaded the Hidden Markov Model (HMM) of the Hsp70 domain from the Pfam database (pfam: PF00012) in order to find candidate Hsp70 protein sequences [22]. We downloaded the additional HMM maps from the PANTHER classification system for Hsp12a and Hsp12b (PTHR14187: SF46 and PTHR14187: SF3). HMMER 3.2.1 was used to identify the Hsp70s from the *M. labio* genome [23]. The Hsp70 domain was screened for their presence in the acquired protein sequences using the NCBI Conserved Domain Database (CDD, https://www.ncbi.nlm.nih.gov/cdd/; *E*-value < 0.001; other settings that are in the default state) (accessed on 11 December 2022) and the Pfam database (http://pfam-legacy.xfam.org/) (accessed on 11 December 2022).

### 2.2. Phylogenetic Analysis of the Hsp70 Genes

The Hsp70 gene, known in vertebrates such as crustaceans and in mammals, is used to identify *M. labio*. The representative species were humans (*Homo sapiens*), platypuses (*Ornithorhynchus anatinus*), mice (*Mus musculus*), chickens (*Gallus gallus*), medakas (*Oryzias latipes*), large yellow croakers (*L. crocea*), zebrafish (*Danio rerio*), torafugu (*Takifugu rubripes*), Chinese soft-shelled turtles (*Pelodiscus sinensis*), Nile tilapias (*Oreochromis niloticus*), African clawed frogs (*Xenopus laevis*), lizards (*Anolis carolinensis*), and swimming crabs (*Portunus trituberculatus*). According to Song et al. [24], the query sequences were obtained from Ensembl (http://asia.ensembl.org/index.html) (accessed on 13 December 2022), NCBI (http://www.ncbi.nlm.nih.gov) (accessed on 13 December 2022), and UniProt (http://www.uniprot.org) (accessed on 13 December 2022). The S1 file in the references [17] was where the *P. trituberculatus* sequences were obtained. A Multiple Protein Sequence Alignment (MUSCLE) program was used to conduct multiple sequence alignments (other options default). RAxML (version 8.2.12) [25] was used to construct the maximum likelihood (ML) phylogenetic tree (1000 bootstrap replicates).

### 2.3. Physicochemical Properties, Gene Structure Analysis, Motif Analysis, and Chromosome Localization of Hsp70 Genes

The ExPASy program (https://web.expasy.org/protparam/) (accessed on 15 December 2022) was used to forecast the basic physicochemical properties of Hsp70 proteins, such as isoelectric points (pI) and theoretical molecular weights (kDa). The Multiple EM for Motif Elicitation (MEME) software (version 5.4.1) [26] was used to identify the conserved motifs of the *M. labio* Hsp70s. For the MEME analysis, the parameters included a width range from 6 to 50 and a restriction of zero or one site per sequence; the default values were assigned to all other parameters. The structure and chromosomal position of the *M. labio* Hsp70s were examined using TBtools v1.09861 software [27] in accordance with the genome annotation file. Using the default settings, DOG 2.0 [28] was utilized to visualize the protein structure. Jalview (version 2.11.1.5) [29] was used for multiple sequence alignment, and homologous gene visualization was used in order to confirm *hsc70*-like names.

### 2.4. Analyzing the Subcellular Localization and Predicting Signal Peptides of Hsp70 Proteins

SOPMA (http://npsa-pbil.ibcp.fr/cgi-bin/npsa_automat.pl?page=npsa_sopma.html) (accessed on 20 December 2022) was utilized to forecast the secondary structure of the proteins. Based on the chromosomal location given in the genomic annotation file, the subcellular localization of Hsp70 proteins was predicted using the WoLF PSORT program (https://wolfpsort.hgc.jp/) (accessed on 21 December 2022). The SignalP 6.0 program (https://services.healthtech.dtu.dk/service.php?SignalP) (accessed on 25 December 2022) was utilized to forecast the Hsp70 protein’s signal peptide.

### 2.5. Expression and Analysis of Hsp70 Genes

We gathered twenty-eight healthy, whole females with comparable sizes and developmental stages from Zhoushan Aquatic Products Market in Zhejiang Province. To lessen the impact of other factors on RNA-seq, all *M. labio* individuals were momentarily placed into artificial saltwater (25 psu) and acclimated during a 24 h period [11]. For 48 h, seven snails in each of four 500 mL beakers were subjected to different levels of polystyrene (PS) PS-NPs. In the experiment, there were three groups: L, M, and H, which were exposed to PS-NPs concentrations of 0.1, 1, and 10 mg/L, respectively; the control group (C) did not undergo exposure to PS-NPs, with 7 snails in each group. Polystyrene nanoplastics (2.5% *w*/*v*, 80 nm) suspension was prepared by the Base Line Chromtech Research Centre (Tianjin, China), and then diluted with saline (25 psu) to prepare three concentrations of suspension.

After the stress was over, dead and diseased snails were removed, and three complete, healthy snails were collected from each group, and their muscles were taken. Individual variance might have been reduced in stress treatments by using a pooling sample method. Then, they were put in RNA buffer, −80 °C standby. After building the sequencing library in accordance with the manufacturer’s kit, the samples were sequenced using the Illumina Hiseq 2500 platform. Out of the 14 expressed genes, 10 genes were chosen at random for qRT-PCR confirmation. Three bioreplications of each cDNA template were performed using 18s as the reference gene.

Bowtie2 was used to build an index from transcripts to the coding sequence diagram. RNA-Seq expectation maximization (RSEM) was used to evaluate the expression of differential genes [30]. RSEM 1.3.1 and Bowtie2 were used to align reads to quantify the expression level. Based on the length of the gene, the predicted number of fragments per kilobase of transcript sequence per million base pairs sequence (FPKM) of the Hsp70s was determined as the unit of expression. Normalized data were used to compute the fold changes and results of gene comparison. The log_2_-based fold change (log_2_FC) were then computed.

In order to investigate the particular gene expansion of this species, we performed a selective test of *M. labio hsc70*-like gene pairs from the developmental relationship and expression profile of Hsp70s. KaKs_Calculator2.0 software [31] was used to calculate the rates of synonymous (Ks) and nonsynonymous (Ka) substitutions and their ratios.

## 3. Results

### 3.1. Genome-Wide Identification and Sequence Analysis of Hsp70 Genes in M. labio

We obtained 21 Hsp70 candidate proteins from the HMM map of pfam Hsp70 domain (PF00012). Annotation file confirmation, CDD searches, and Pfam scans led to the rejection of six candidate genes (without the full Hsp70 domain). In *M. labio*, the Hsp70 gene family consists of 15 Hsp70 protein sequences. Table 1 presents the basic characteristics (gene name, protein length, coding-sequence length, Hsp70 domain region, isoelectric point, molecular weight, and genome location) of Hsp70 genes of *M. labio*. These Hsp70 genes encoded proteins with amino acids (aa) ranging from 591 to 967, while their CDSs varied in length from 1776 to 2904 bp. Based on the anticipated amino acid sequence’s physicochemical characteristics, hyou1 (752 aa) had the longest conserved domain and hspa9l.1 (553 aa) had the smallest. The Hsp70 proteins ranged in projected isoelectric points (pI) from 4.86 (*hyou1*) to 5.92 (*hspa9*), with molecular weights between 64,170.27 and 109,317.80 kDa.

### 3.2. Phylogenetic Analysis of Hsp70 Genes

**Figure 1:**
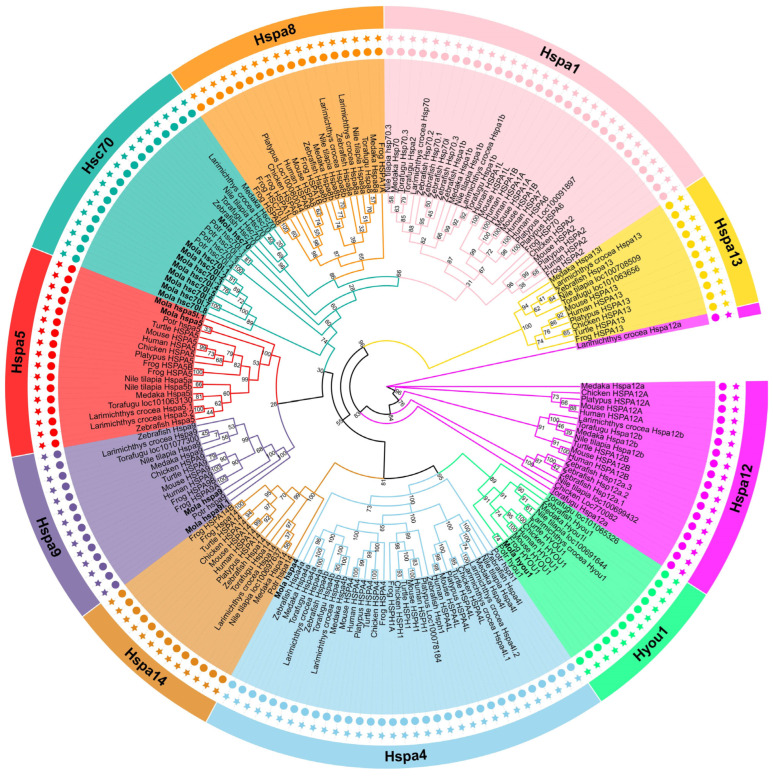
Phylogenetic study of all species’ Hsp70 proteins. *M. labio* Hsp70s are shown in bold. Evolview online website (https://evolgenius.info//evolview-v2/#login) (accessed on 15 December 2022) to beautify.

The *M. labio* Hsp70 gene family members were given names based on branches of the evolutionary tree. All of *M. labio* Hsp70 members were clearly divided into different systems and grouped with proteins from other species in the phylogenetic ML tree. The ML tree clearly showed one distinct cluster in *M. labio* (Figure 1). The cluster included nine copies of *M. labio* Hsc70 genes (*hsc70*-like), suggesting a mollusk-specific gene expansion. Nine copies of *M. labio* Hsc70 genes (*hsc70*-like) were strongly orthologous to the *hsc70* genes of *D. rerio*, *L. crocea*, *O. latipes*, *T. rubripes*, *O. niloticus*, and *P. trituberculatus*. The zebrafish Hsps nomenclature criteria were followed in the naming of the Hsp70 genes (Figure 1).

### 3.3. Analysis of Motif, Structure, and Conserved Domain

**Figure 2:**
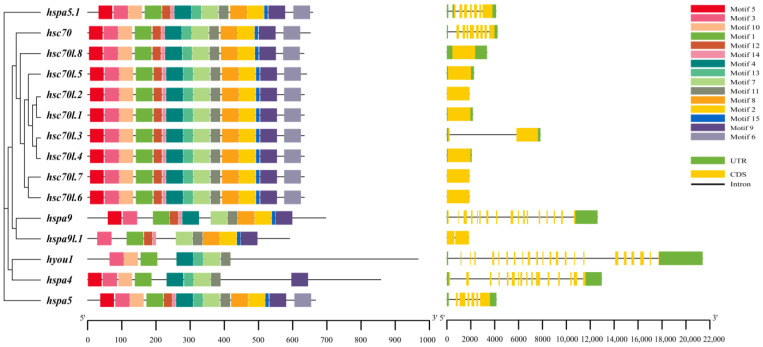
Distribution of *M. labio* Hsp70 protein conserved motifs. Motif displayed with TBtools. The structural characteristics of 15 Hsp70s. The green lines represent the untranslated region (UTR), while the yellow lines represent the coded region (CDS). The black line denotes introns.

Fifteen conserved motifs of *M. labio* in protein sequences were found after employing MEME to investigate and analyze them (Figure 2). The Hsp70 gene family has one to fifteen motifs, which are referred to as motifs 1–15. The recently duplicated hsc70 homologs’ protein structures in our study displayed a similar motif arrangement (Figure 2).

Although Hsp70 genes were assigned into different groups, they had motifs 1, 3, 7, and 11 of the same kind and distribution. *hsc70*-like genes had all the same conservative motifs, namely, motifs 1–15. To learn more about this gene family’s evolutionary conservation, we also investigated the gene structures of 15 Hsp70 genes. In these Hsp70 genes, the quantity of CDS-intron structures differed significantly, reflecting the evolution of gene families (Figure 2). The number of CDS varied from 1 to 22 in these genes (Figure 2). The quantity of CDS separated these genes into two patterns: pattern 2 has more than two CDS, while pattern 1 only has one or two CDS. Pattern 1 included *hsc70l.1*–*hsc70l.8,* and *hspa9l.1*. *hsc70*, *hspa9*, *hspa5*, *hspa5l.1*, *hspa4*, and *hyou1* were all present in Pattern 2.

**Figure 3:**
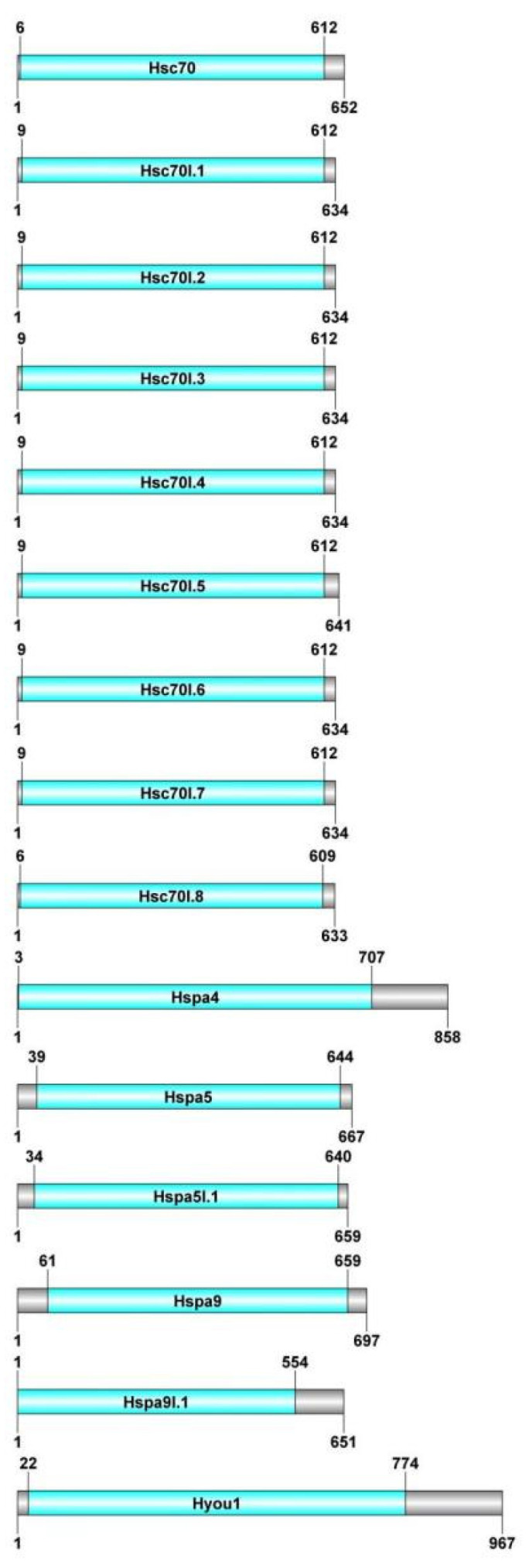
Predictions of conserved domains for 15 Hsp70 proteins. The length of each protein sequence is shown using gray bars, while blue boxes indicate conserved domains. Protein-structure visualization was completed using DOG 2.0.

Using CDD and Pfam, the structures of the Hsp70s were further described (Figure 3). A highly conserved region at the N terminus, between 1 and 774 aa, was present in all Hsp70 proteins. Their existence acted as evidence that they were, in fact, Hsp70 proteins.

### 3.4. Secondary Structure Prediction, Subcellular Localization, and Analysis of Hsp70 Proteins

Among the 15 Hsp70 proteins, α helices and random coils are the most important secondary structures. According to Table 2, α helices made up 40.95–47.05%, β turns, 3.03–7.89%, random coils, 29.44–38.81%, and extended strands, 14.10–21.32%.

Hsp70 proteins are expressed in the mitochondria, cytoplasm, nucleus, and endoplasmic reticulum in *M. labio*. The majority of Hsc70-like proteins were expressed in the cytoplasm (Table 2). The endoplasmic reticulum expressed the proteins Hspa5 and Hyou1. The proteins Hspa9 and Hspa5l.1 were expressed in the mitochondrion. Within the nucleus, Hspa4 and Hsc70l.5 proteins were expressed.

### 3.5. Analysis of Protein Signal Peptides Predictively

**Figure 4:**
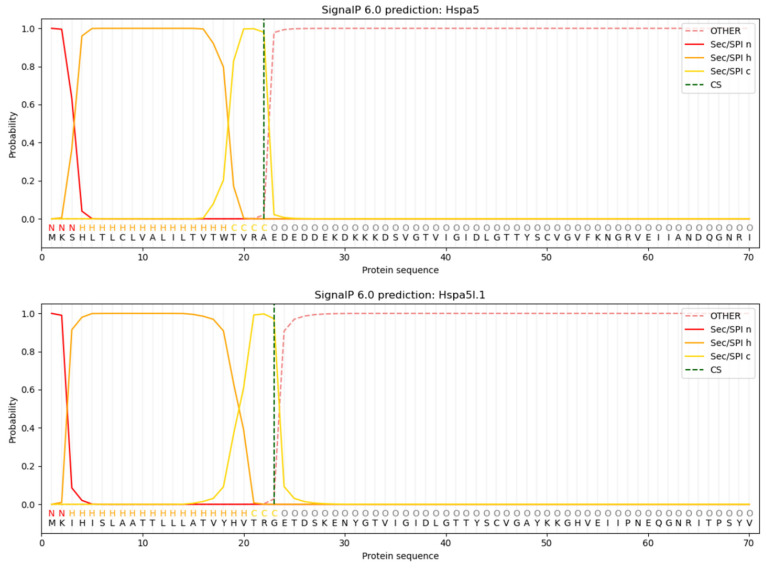
Hsp70 protein signal peptide prediction analysis (Hspa5, Hspa5l.1).

Only Hspa5 and Hspa5l.1 were predicted and preliminarily identified as secreted proteins in Hsp70 protein in *M. labio*. The 22nd alanine (A) had the highest raw cleavage site score (CS) for Hspa5, with a value of 0.9796. Hspa5l.1 CS reached its maximum value of 0.9722 on the 23rd glycine (G). Based on the SP value, Hspa5 and Hspa5l.1 had signal peptides, with lengths of roughly 22 and 23 amino acids, respectively (Figure 4).

### 3.6. Chromosomal Location Analysis of Hsp70s

**Figure 5:**
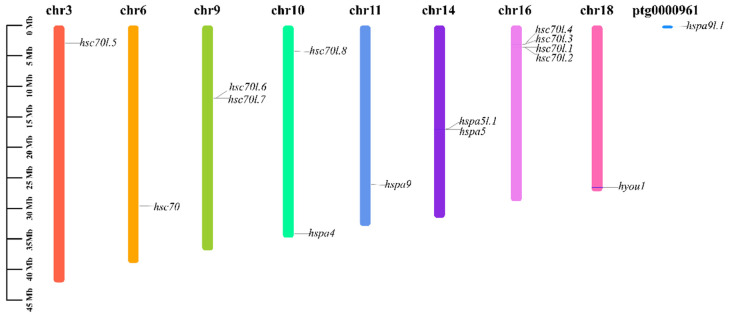
Hsp70 genes distribution in *M. labio* chromosomes and contigs. At the top of every vertical color bar was the number of chromosomes.

Based on chromosomal localization analysis, the 15 members of the Hsp70 gene family are distributed on eight chromosomes. As shown in Table 1, *hsc70l.1-hsc70l.4*, *hsc70l.6*, and *hsc70l.7*; *hsc70l.8* and *hspa4*; and *hspa5* and *hspa5l.1* were distributed on the same chromosomes. The density was highest on chromosome 16, which contained four hsc70-like genes (Figure 5).

### 3.7. Expression of the Hsp70 Genes in M. labio Muscles under NP Stress

**Figure 6:**
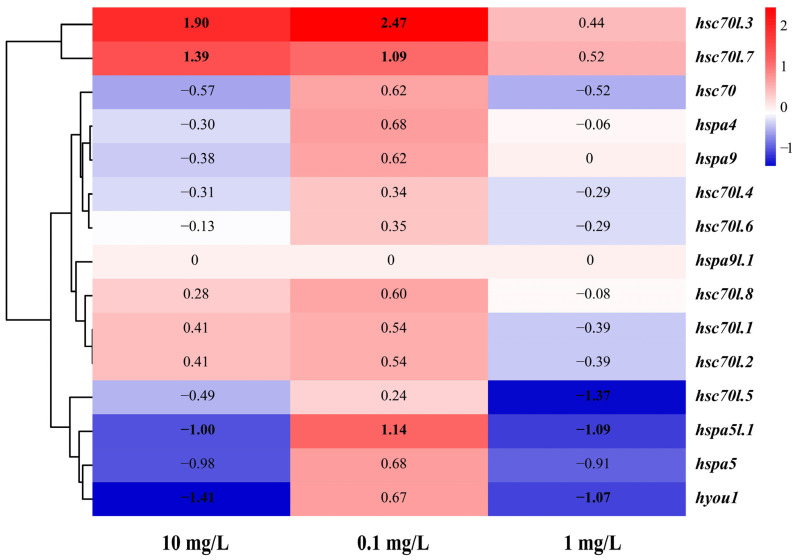
Expression profiles of *M. labio* Hsp70 gene family in muscles. Every gene’s expression level was compared to the control data (0 mg/L). A heat map was made using FPKM measurements and a log_2_-based fold change. FPKM stands for fragments per million exons per kilobase. Bold indicates the significantly genes (*p* < 0.05; log_2_FC > 1.0 or log_2_FC < −1.0).

After *M. labio* were acutely exposed to various NP concentrations, the study’s findings revealed that of the 15 hsp70 genes, 14 were expressed in muscle tissue, but hspa9l.1 was not (Table 3, Figure 6). Five *hsp70* genes among them were significantly involved in responses to different concentrations of NPs (log_2_FC > 1.0 or log_2_FC < −1.0). Under group L (NP stress treatment at 0.1 mg/L), three genes were significantly upregulated (*hsc70l.3*, *hsc70l.7*, *hspa5l.1*; log_2_FC: 2.47, 1.09, 1.14), and the rest were slightly upregulated. Under group M (NP stress treatment at 1 mg/L), two *hsp70* genes were significantly upregulated (*hsc70l.3*, *hsc70l.7*; log_2_FC: 1.90, 1.39), and two were significantly downregulated (*hspa5l.1*, *hyou1*; log_2_FC: −1.0, −1.41). Under group H (NP stress treatment at 10 mg/L), three *hsp70* genes were significantly downregulated (*hsc70l.5*, *hspa5l.1*, *hyou1*; log_2_FC: −1.37, −1.09, −1.07), and two genes were slightly upregulated (*hsc70l.3*, *hsc70l.7*; log_2_FC: 0.44, 0.52). The remaining *hsp70* genes were slightly downregulated.

### 3.8. Selection Test on Duplicated Hsp70 Genes

According to the Ka and Ks and their ratios in order to comprehend the selection pressure and species-specific gene expansion, for *hsc70l.2-hsc70l.1*, *hsc70l.3-hsc70l.4*, and *hsc70l.6-hsc70l.7* couples, the corresponding Ka/Ks ratios were 0, 0.1244, and 0.0495. The Hsp70 genes pairs’ Ka/Ks values were all less than 1.0, suggesting that purifying selection was applied to these genes during the evolutionary process (Table 4).

## 4. Discussion

Hsp70s are crucial for responses to both biotic and abiotic stressors. Recent years have seen a large-scale effort to identify Hsp70 genes across the genome in insects [32], plants [33], mollusks [20], fishes [24], and mammals [34]. In the present study, 15 Hsp70 genes were found, including two single-copy genes (*hspa4*, *hyou1*) and three pairs of genes with duplicates (*hsap5*, *hspa5l.1*; *hspa9*, *hspa9l.1*; *hsc70*-like genes). The number of Hsp70 genes in *M. labio* differed from those of vertebrates, which do not have *hspa2*, *hspa8*, *hspa12*, *hspa13*, *hspa14*, *and hsph1* (Table 1). In contrast to the members of bivalves, whether *hspa12*, *hspa13*, *and hspa14* genes were truly missing from the *M. labio* genome remains unclear. Nine copies of duplicate *hsc70*-like genes were discovered in *M. labio*, these genes’ structures and motifs are similar to the function. Similar conserved domains were also present in the amino acids of *hsc70*-like genes 6 to 612, suggesting that they may have comparable roles, as supported by evidence from the homologous protein sequence alignment. According to the subcellular localization of Hsp70 genes, these genes are dispersed throughout the cell, including the cytoplasm, mitochondria, endoplasmic reticulum, and nucleus [35]. Prior research has documented the critical functions of cytosolic Hsp70s in both stressful and non-stressful environments [36]. Gene pairs can be found on various chromosomes based on the members and chromosomal location of Hsp70 genes and a phylogenetic tree in *M. labio*. It is important to note that gene duplication might be regarded as fragmented once gene pairs are found on distinct chromosomes. Furthermore, genes duplicated on the same chromosome are referred to as tandem duplication [37]. 

Gene duplications are traditionally considered a significant evolutionary source of new protein functions; they are present in all living forms and serve as a foundation for functional innovation. The common selection of existing genes can produce new functions, and new transcriptional regulatory sites can be developed to alter gene expression [38]. In the present work, local gene duplications are an important component of the mechanism hsp70 genes’ amplification in *M. labio* because all amplified *hsc70*-like genes (9 copies) existed as tandem gene clusters. Various species have various numbers and kinds of duplicated genes, suggesting that these duplications may have separate origins [39]. Gene duplications and tandem duplications have been previously described in teleost fishes, echinoderms, and bivalve species as well as in crustaceans [39,40]. For example, previous studies indicated the expansion of the Hsp70 genes from Hspa4 (4 copies) in *L. crocea*, Hspa1 in *B. pectinirostris* (7 copies), Hspa12 in *C. gigas*; *P. yessoensi* (73 copies and 57 copies), Hsc70 in *P. trituberculatus* and *M. labio* (4 and 9 copies, respectively).

This replication event revealed the dynamic evolution of the genome, resulting in a series of complex physiological mechanisms, enabling intertidal animals to have their own unique way of life to respond to stresses. Numerous common instances of particular gene sets expanding within particular lineages are linked to modifications in morphological, behavioral, or physiological characteristics. For instance, the *hsp70* (5 copies) gene expression profiles in sea cucumbers *Apostichopus japonicus* during aestivation showed patterns distinct to both tissues and individuals [17]. *P. yessoensis* has large numbers of Hsp gene (*hspa12*) expansion to deal with defensive mechanisms against various environmental stresses [20]. Oysters express a large number of Hsps genes during low-tide stress [21].

During evolution, gene replication is an important mechanism for genome-wide duplication (WGD) or single-gene duplication (SSD) to amplify gene families [41]. Therefore, the repeated selection strategy is predicted by calculating the Ka/Ks ratio of gene pairs [42]. Moreover, purifying selection was observed in the selection test conducted on the duplicate hsp70 genes pairs, indicating that these genes can go through sub-functionalization and result in functional redundancy in *M. labio.*

Different types of stresses have different effects on the expression profile of Hsp70s, such as thermal stress, low salinity, and high ammonia. In muscle tissue under NP stress, 14 out of the 15 Hsp70s were expressed. The significantly expressed Hsp70s were found from the *Hyou1* gene and the mollusk-specific expansion of the *Hsc70*, *Hspa5* subfamily. Hence, to evaluate the expression regulation of *M. labio* Hsp70s in response to NP stress, this study concentrated on genes that were significantly expressed. The expression patterns of various Hsp70s varied under NP stress, potentially due to the diverse physiological effects caused by different concentrations of NPs. Under normal circumstances, the expression of several Hsp70 gene members was incredibly low, but in response to stressors, it increased dramatically. Comparable findings showed that following exposure to biotic and abiotic stressors, the *hsp70* gene was significantly expressed in *L. crocea* [15], *B. pectinirostris* [14], *P. yessoensis* [20], and *P. trituberculatus* [16]. 

*hsc70l.3* and *hsc70l.7* were significantly upregulated in the muscle after treatment with 0.1 and 1 mg/L NPs; however, *hsc70l.5* was significantly downregulated after 10 mg/L NP stress treatment. Strong expression of *hsc70l.3* and *hsc70l.7*, at 0.1 and 1 mg/L, shielded cells from NP-induced misfolding and harm [39]. The production of Hsc70l.3 and Hsc70l.7 progressively increased, most likely due to the requirement for more Hsc70l.3 and Hsc70l.7 proteins, which encouraged aberrant proteins to renaturate [16,43]. When exposed to 10 mg/L of NP stress, the quantity of damaged proteins increased [16]. With increasing concentrations of NPs, we speculated that the synthesis and degradation of misfolded proteins were inhibited by high NP stress [15]. It may result in intracellular hypoxia at 1 and 10 mg/L NP stress, which would slow down the rates of membrane transport and enzymatic activities. Hspa5l.1 was predicted and tentatively identified as a secreted protein present in the mitochondrion with a transmembrane domain and a signal peptide. In this investigation, protein synthesis in mitochondria was suppressed and expression decreased with increasing NP concentrations. Thus, the folding and secretion of proteins may be impacted by the expression of the *hspa5l.1* gene. 

## 5. Conclusions

Using bioinformatics, the toxicological response of *M. labio* to NP stress was investigated for the first time. Fifteen Hsp70 genes were found to be present when exposed to NPs. Nine of the 15 Hsp70s genes belong to the Hsc70 subfamily and have undergone mollusk-specific gene amplification. Hsp70s’ expression was shown to be regulated; upon exposure to NPs, a few inducible genes, including *hsc70l.3*, *hsc70l.5*, *hsc70l.7*, *hspa5l.1*, and *hyou1*, were either up or downregulated, and they were from the Hyou1, Hspa5 sub-family, and the mollusk-specific enlarged Hsc70 sub-family. The NP-stress response may be the result of the evolution of tandem repeats of Hsp70s. These data ought to be helpful for both studying the evolutionary history of mollusk species and comprehending the roles played by Hsp70s. Further investigation of the activities of mollusk Hsp70s will advance knowledge of the defense strategies mollusks employ to fend off a variety of environmental threats.

## Figures and Tables

**Table 1:** The information of 15 Hsp70 genes identified in *M. labio* was summarized.

No.	Gene Name	Gene ID	CDS * Length (bp)	Protein Length (aa)	Hsp70 Domain Location (aa)	MW * (kDa)	PI *	Chromosome	Location
1	*hsc70*	Mlab0082280.1	1959	652	6-612	71,385.57	5.24	chr6	29612442:29616687
2	*hsc70l.1*	Mlab0108740.1	1905	634	9-612	69,628.69	5.51	chr16	3599462:3601643
3	*hsc70l.2*	Mlab0108730.1	1905	634	9-612	69,628.69	5.51	chr16	3603044:3604948
4	*hsc70l.3*	Mlab0108940.1	1905	634	9-612	69,483.42	5.39	chr16	3103368:3111202
5	*hsc70l.4*	Mlab0108950.1	1905	634	9-612	69,582.56	5.45	chr16	3099140:3101222
6	*hsc70l.5*	Mlab0079900.1	1926	641	9-612	70,145.10	5.64	chr3	2896965:2899237
7	*hsc70l.6*	Mlab0033790.1	1905	634	9-612	69,631.49	5.56	chr9	11909551:11911455
8	*hsc70l.7*	Mlab0033800.1	1905	634	9-612	69,608.45	5.51	chr9	11912615:11914519
9	*hsc70l.8*	Mlab0114000.1	1902	633	6-609	69,727.77	5.55	chr10	4284735:4288100
10	*hspa4*	Mlab0150810.1	2577	858	3-707	96,570.18	5.18	chr10	34117494:34130454
11	*hspa5*	Mlab0102200.1	2004	667	39-644	73,546.11	5.03	chr14	17073207:17077361
12	*hspa5l.1*	Mlab0102100.1	1980	659	34-640	72,963.32	5.38	chr14	16986162:16990290
13	*hspa9*	Mlab0147970.1	2094	697	61-659	76,155.32	5.92	chr11	26113170:26125780
14	*hspa9l.1*	Mlab0204400.1	1776	591	1-554	64,170.27	4.86	ptg000096l	225489:227341
15	*hyou1*	Mlab0200990.1	2904	967	22-774	109,317.80	5.48	chr18	26557938:26579334

* CDS: coding sequence. MW: molecular weight. PI: isoelectric points.

**Table 2:** Hsp70 proteins’ secondary structure and subcellular localization prediction in *M. labio*.

Protein	α Helix	β Turn	Random Coil	Extended Strand	Subcellular Location Prediction
Hsc70	40.95%	7.21%	33.44%	18.40%	Cytoplasm
Hsc70l.1	41.48%	7.89%	31.55%	19.09%	Cytoplasm
Hsc70l.2	41.48%	7.89%	31.55%	19.09%	Cytoplasm
Hsc70l.3	41.64%	7.41%	32.18%	18.77%	Cytoplasm
Hsc70l.4	41.64%	7.41%	32.02%	18.93%	Cytoplasm
Hsc70l.5	42.28%	6.40%	32.61%	18.72%	Nucleus
Hsc70l.6	42.43%	7.10%	31.39%	19.09%	Cytoplasm
Hsc70l.7	42.90%	7.10%	31.23%	18.77%	Cytoplasm
Hsc70l.8	42.81%	6.79%	32.07%	18.33%	Cytoplasm
Hspa4	44.06%	3.03%	38.81%	14.10%	Nucleus
Hspa5	43.03%	7.20%	31.63%	18.14%	Endoplasmic reticulum
Hspa5l.1	42.49%	6.98%	31.26%	19.27%	Mitochondrion
Hspa9	44.19%	7.32%	29.56%	18.94%	Mitochondrion
Hspa9l.1	42.47%	6.77%	29.44%	21.32%	Cytoplasm
Hyou1	47.05%	4.65%	33.92%	14.37%	Endoplasmic reticulum

**Table 3:** *M. labio* Hsp70 genes’ expression in the muscle at NP stress was measured using log_2_FC and FPKM. Bold indicates the prominent genes (*p* < 0.05; log_2_FC > 1.0 or log_2_FC < −1.0).

Gene	log_2_FC		
	0.1 mg/L	1 mg/L	10 mg/L
*hsc70*	0.62	−0.57	−0.52
*hsc70l.1*	0.54	0.41	−0.39
*hsc70l.2*	0.54	0.41	−0.39
*hsc70l.3*	2.47	1.90	0.44
*hsc70l.4*	0.34	−0.31	−0.29
*hsc70l.5*	0.24	−0.49	−1.37
*hsc70l.6*	0.35	−0.13	−0.29
*hsc70l.7*	1.09	1.39	0.52
*hsc70l.8*	0.60	0.28	−0.08
*hspa4*	0.68	−0.30	−0.06
*hspa5*	0.68	−0.98	−0.91
*hspa5l.1*	1.14	−1.00	−1.09
*hspa9*	0.62	−0.38	0.00
*hspa9l.1*	0.00	0.00	0.00
*hyou1*	0.67	−1.41	−1.07

**Table 4:** Ka/Ks values of homologous Hsp70 genes’ pairs. Ka: nonsynonymous substitution rate; Ks: synonymous substitution rate.

Gene-Pair	Ka	Ks	Ka/Ks
*hsc70l.2*–*hsc70l.1*	0	0	0
*hsc70l.3*–*hsc70l.4*	0.0008	0.0064	0.1244
*hsc70l.6*–*hsc70l.7*	0.0007	0.0139	0.0495

## Data Availability

The raw sequence data reported in this paper have been deposited in the Genome Sequence Archive in the National Genomics Data Center, China National Center for Bioinformation/Beijing Institute of Genomics, Chinese Academy of Sciences (GSA: CRA012447) that is publicly accessible at https://ngdc.cncb.ac.cn/gsa (accessed on 11 December 2022). The identified Hsp70 genes of *M. labio* have been uploaded as supplementary document.
